# Supplementary material for: Transarterial Chemoembolization Outperforms Radioembolization in Early- and Intermediate-Stage Hepatocellular Carcinoma: A Multicenter Retrospective Study
Source: Cancers (Basel). 2025 Jul 7;17(13):2254. doi: 10.3390/cancers17132254 (PMC12249255; doi:10.3390/cancers17132254)
Supplement: Supplementary file 1 [file cancers-17-02254-s001.zip › cancers-3716259-supplementary.pdf]

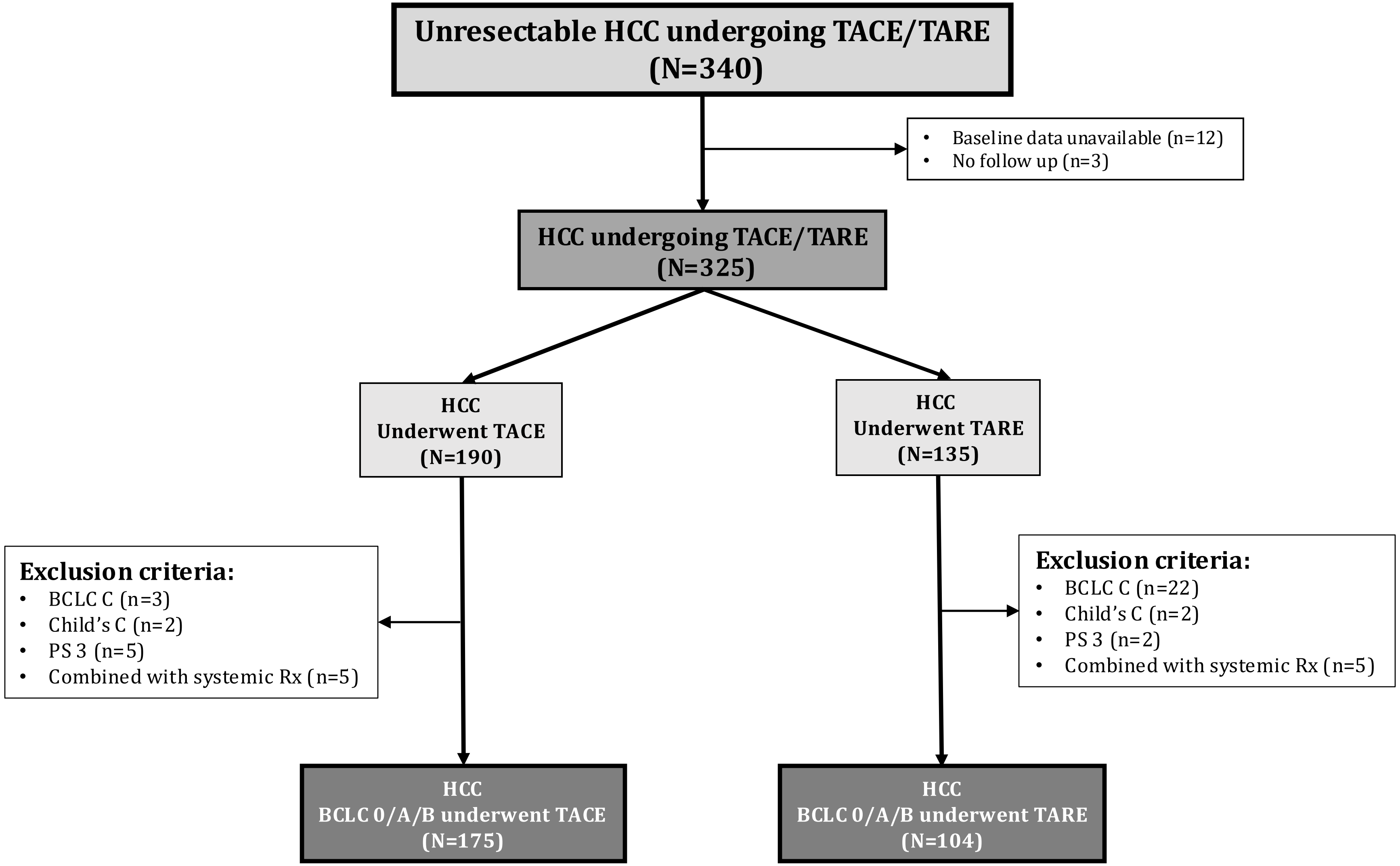

Supplementary Figure S1: Study schematic showing patient disposition in both treatment groups

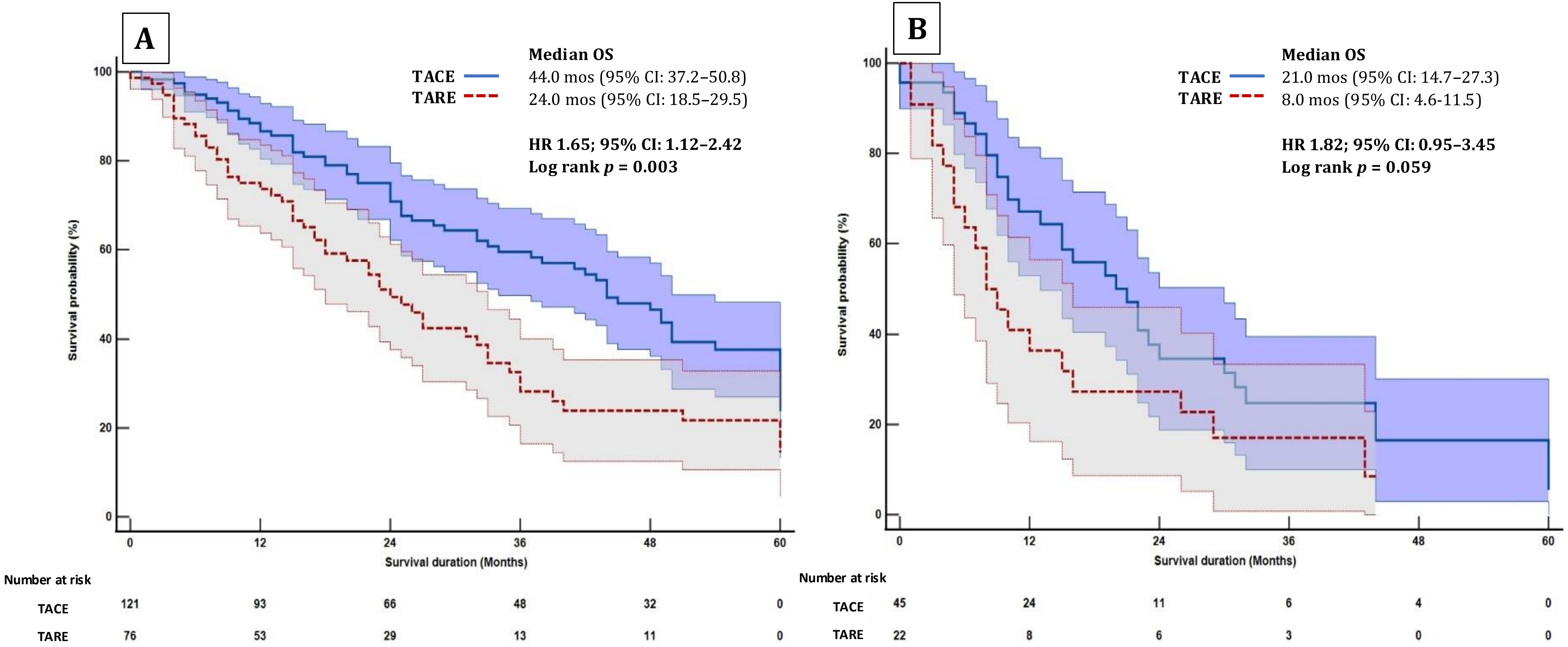

Supplementary Figure S2: Survival analysis of patients undergoing transarterial chemoembolization and radioembolization in sub-groups of **A)** Child-Pugh A; and **B)** Child-Pugh B

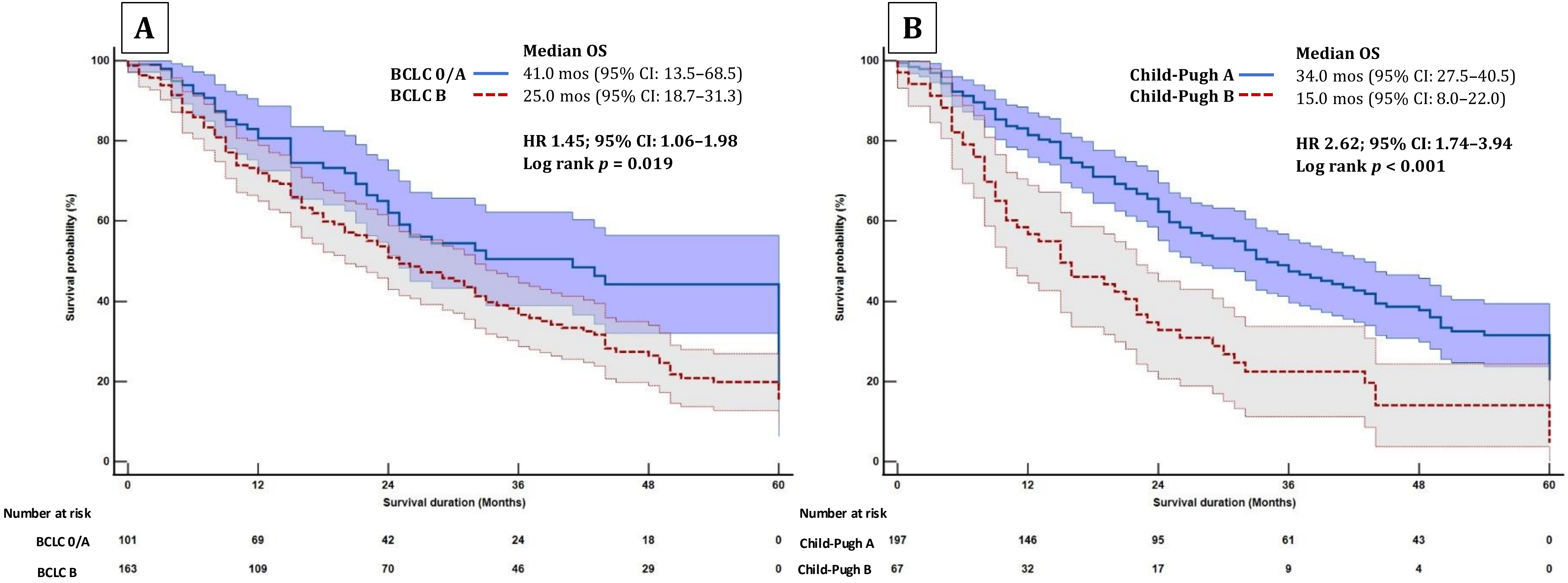

Supplementary Figure S3: Survival analysis of overall cohort of patients as stratified by **A)** BCLC stage and **B)** Child-Pugh stage

Supplementary Table S1. Summary of safety events (>5%) among patients treated with TACE and TARE.

Adverse event		Total n = 279	TACE n = 175	TARE n = 104	*p*
**Abdominal pain**		33 (12.6)	27 (15.8)	6 (6.0)	0.011
**Fever**		10 (3.8)	5 (5.0)	5 (2.0)	0.221
**Vomiting**		13 (5.0)	10 (6.3)	3 (3.0)	0.381
**ALT elevation**		45 (16.1)	35 (20.0)	10 (9.6)	0.028
	>2 ULN	31 (12.1)	24 (15.4)	7 (6.9)	0.050
	>5 ULN	14 (5.5)	11 (7.1)	3 (3.0)	0.260
**Post-embolization syndrome**		13 (5.0)	10 (6.2)	3 (3.0)	0.381
**Hepatic decompensation** *		51 (18.3)	24 (13.7)	27 (18.3)	0.010
	Variceal bleeding	4 (1.4)	1 (0.6)	3 (2.9)	0.148
	Encephalopathy	17 (6.1)	9 (5.1)	8 (7.7)	0.611
	Ascites	43 (15.4)	19 (10.9)	24 (23.1)	0.006
**Serious adverse events**		5 (1.8)	3 (1.7)	2 (1.9)	-
	Cholecystitis	2 (0.7)	2 (1.1)	0 (0)	-
	Gastric ulcerations	1 (0.4)	0 (0)	1 (1)	-
	Acute liver failure	2 (0.7)	1 (0.6)	1 (1)	-

Data presented as n (%); * hepatic decompensation could have >1 decompensating event in the same patient. TACE, transarterial chemoembolization; TARE, transarterial radioembolization; ALT, alanine aminotransferase; ULN, upper limit of normal.
